# Missed opportunities: Do states require screening of children for health conditions that interfere with learning?

**DOI:** 10.1371/journal.pone.0190254

**Published:** 2018-01-17

**Authors:** Delaney Gracy, Anupa Fabian, Corey Hannah Basch, Maria Scigliano, Sarah A. MacLean, Rachel K. MacKenzie, Irwin E. Redlener

**Affiliations:** 1 Children’s Health Fund, New York, NY, United States of America; 2 Department of Public Health, William Paterson University, New York, NY, United States of America; 3 Department of Epidemiology, Mailman School of Public Health, Columbia University, New York, NY, United States of America; 4 Department of Health Policy and Management, Mailman School of Public Health, Columbia University, New York, NY, United States of America; Karolinska Institutet, SWEDEN

## Abstract

Strong evidence supports the existence of Health Barriers to Learning (HBLs)—health conditions that when untreated or unmanaged can interfere with a child’s ability to learn and succeed in school. These HBLs include vision and hearing deficits, uncontrolled asthma, mental and behavioral problems, dental pain, persistent hunger, and the effects of lead exposure. However, 19% of US children aged 6 to 11 did not receive their annual checkup in the past year. School requirements for health screenings can help identify children with HBLs. This study explores which states require health screening for children in elementary school, and the extent to which the 7 HBLs are included.

**Methods:**

Investigators reviewed websites of state departments of health and education, and legislation for all 50 states and DC. For states with mandated screenings and a required form, investigators applied structured analysis to assess HBL inclusion.

**Results:**

No state mandated that schools require screening for all 7 HBLs. Less than half (49%) required comprehensive school health examinations and only 12 states plus DC required a specific form. Of these, 12 of the forms required documentation of vision screening, 11 of hearing screening, and 12 of dental screening. Ten forms asked about asthma and 9 required documentation of lead testing. Seven asked about general well-being, emotional problems, or mental health. None addressed hunger. When including states without comprehensive school health examination requirements, the most commonly required HBL screenings were for vision (80% of states; includes DC), hearing (75% of states; includes DC) and dental (24% of state; includes DC).

**Conclusion:**

The lack of state mandated requirements for regular student health screening represents a missed opportunity to identify children with HBLs. Without state mandates, accompanying comprehensive forms, and protocols, children continue to be at risk of untreated health conditions that can undermine their success in school.

## Introduction

The years that a child spends in primary school, typically pre-K or Kindergarten to 5th or 6th grade and spanning ages 4 through 12, are critical in his or her health, psychological, social, and educational development [1,2]. All of these domains are interdependent and fundamental building blocks of future quality of life and social and financial stability [3,4].

### Health Barriers to Learning

Extensive research exists linking health and a child’s ability to perform optimally in school [5,6]. For children in primary school, specific conditions with a strong evidence base, referred to here as “Health Barriers to Learning” (HBLs), include: vision and hearing deficits, uncontrolled asthma, mental and behavioral problems, dental pain, persistent hunger, and the effects of lead exposure. Left untreated, these HBLs can adversely affect a child’s ability to receive and process information, pay attention in class, ability and motivation to learn, attendance, academic performance, and even his or her chances of graduating from high school [7]. The evidence base for these specific health barriers is described in detail in the 2017 literature review, *Health Barriers to Learning*: *The Prevalence and Educational Consequences in Disadvantaged Children* [7], but are summarized below.

### Vision

Though estimates of prevalence vary depending on population and specific vision issues, studies show that 22%-30% of children fail vision screening [8–11]. About 80% of learning occurs through visual tasks such as reading, writing and using computers; studies conclude that uncorrected vision problems impede a child’s ability to read and that correction of the problem improved performance [12–14].

### Hearing

About 1% of children entering school are estimated to have hearing loss [15]. However, concerns are increasing about noise-induced damage, and studies have shown higher rates of hearing loss in adolescence, especially when high frequencies are added to screening tests [16]. Even minimal uncorrected hearing loss is associated with increased risk of poor performance on educational tests; higher rates of dysfunction in speech, language, and behavior; and higher rates of social emotional difficulties, including lower self-esteem [17–19].

### Asthma

While asthma affects about 9% of school-age children nationally, rates as high as 30% have been shown in high-risk minority groups and high-poverty neighborhoods [20]. Poorly controlled asthma has been shown to negatively affect school performance through decreased school readiness, increased absences, and sleep deprivation [21–23]. Poorly controlled asthmatics have been shown to miss 18 days of school per year due to asthma, compared to 2 days per year for students whose asthma is well controlled [24,25].

### Mental and behavioral health

In national survey data from 2015, the percentage of children with serious emotional or behavioral difficulties among children ages 4–7 was approximately 4%, compared with children ages 8–10 at 6%, and children ages 11–14 at 8% [26]. Similar surveys have shown that an additional 17% of children ages six to 11 had minor emotional or behavioral difficulties [27]. Particularly strong evidence exists for the negative impact of attention problems on both math and reading scores, and on school dropout [28–30].

### Dental pain

About 37% of children aged 6 to 9 have dental caries, but the number almost doubles, to 69%, for children living in poverty. Approximately 14% of children overall have caries that are untreated, but the numbers exceed 30% in high-risk minority populations [31]. About 4%-7% of students miss school due to dental problems [32,33]. Multiple studies found that children with dental pain are significantly more likely to sleep poorly, miss school, and are less likely to complete all of their required homework [32,34,35]. Several studies have associated dental pain with lower grades [32,34].

### Persistent hunger

According to national survey data, among households with children, about 17% of households experienced food insecurity during the prior year [36]. Chronic hunger in children has been associated with delayed development, higher rates of both internalizing and externalizing mental health problems, and decreased classroom participation [37–39]. Some studies have shown an association with lower math scores and increased need to repeat grades [40,41].

### Effects of lead exposure

Lead exposure primarily affects children under 7 years old, but the effects are significant, and thus extremely relevant to screening in the early years of school entry and attendance. Nationally, about 2.6% of children younger than 7 have elevated blood lead levels, but in high poverty populations living in high-risk areas, studies have shown histories of elevated lead levels in as many as 69% of children studied [42,43]. The term ‘elevated blood lead levels’ refers to the reference value of 5 μg/dL, though research shows that even lower levels can impact cognition and the developing brain [44]. Numerous studies show decreases in IQ, school performance, and reading and math scores as blood lead levels increase in young children [45–48].

Evidence exists that other health and psychosocial conditions impact educational outcomes, most frequently in the content areas of poverty, trauma, adverse childhood experiences, and sleep [49–53]. Because they are often interrelated with the aforementioned conditions, they warrant mention and are included in the discussion, but not included as specific elements in this study. For any child, a specific chronic health condition that impacts cognition, functioning, or attendance can impact their school performance, and is important to be identified and managed. To be most broadly applicable, this study is limited to the elements listed above, which are both common and supported by strong evidence to impact educational outcomes.

### Gaps in health screening for children

Pediatric primary care recommendations for school-age children include an annual well child check with a full history, physical examination, anticipatory guidance (age-appropriate health education to promote wellness and prevent disease and injury), and targeted screenings [54]. Each year, the American Academy of Pediatrics publishes updated, evidence-based/informed recommendations on specific screenings to be performed for children of each age. According to these guidelines, an age-appropriate behavioral health/mental health assessment, a brief screen for hunger [55] and food insecurity, and a physical examination that includes dental are recommended for every annual well child check. Vision screening is recommended at 6 of the 9 annual visits between the ages of 4 and 12; hearing screening is recommended 5 times in this same interval, both with condition-specific risk assessments to be performed at the annual visits in between. Lead screening is recommended earlier in childhood, at ages 1 and 2, but annual risk assessments should be done until age 6, and all children should have had at least one blood test by age 4 [54,56]. There are not widely adopted guidelines for asthma screening in general pediatric populations, but the standard of care is for an annual well child check that includes questions about cough and breathing trouble that, if affirmative, should lead to a more extensive assessment for asthma [57]. For the above schedules, there are additional recommendations for more frequent screening for children living in poverty, those with specific risk factors, and for children who were not fully screened in prior years [56,58,59]. Whereas children do not necessarily need to be screened for all of the HBLs every year, they should be seen and assessed each year to determine which screenings they do need.

If all children received annual well child checks, and all health care providers followed the best-practice guidelines, children *would* be regularly screened for the Health Barriers to Learning through general primary care. However, nationwide 20% of school-aged children—9.8 million children aged 6 to 17—did not receive their well child checks in the past year. Rates of missed well child checks are high among 6 to 11 year olds (19%), children from families below the poverty line (19%), Hispanic children (21%), and uninsured children (44%) [60].

#### Role of schools in facilitating screening

A key strategy to support timely identification and treatment of educationally relevant health conditions in school-aged children is through health screening requirements for school admission, attendance, and activity participation [61]. This can be achieved by school-required health forms completed by the child's usual doctor or community healthcare provider, through screenings at offered at school, or through other types of community partnerships.

#### Role of states in ensuring screening gaps are minimized

There is no federally required school health screening form or set of school health screening requirements. States are able to create standard forms or screening requirements for schools, but are not required to do so [62–64]. There appears to be little consistency around health screening or particular requirements targeting Health Barriers to Learning. This represents a potential gap in the health safety net for children that is also relevant to their performance and at school [65,66].

### Study scope and objectives

In review of the literature, no comprehensive assessments of state level health screening requirements for primary school students were identified that were not restricted to a single health condition [67]. This study’s objectives are to fill that information gap in the following ways: 1) assess the extent to which states mandate schools to require—through school-based or community-delivered services—health screening for children age 4–12, and 2) assess the extent to which the evidence-based HBLs listed above are included when screening is required. This information has the potential to influence policy around requirements for student health screenings, and to provide a base for future studies on cost effectiveness, implementation, and consideration of health conditions to be included in screening requirements.

## Methods

For each state, investigators asked: Does the state mandate that schools require screening of children for all seven of the Health Barriers to Learning: vision and hearing deficits, uncontrolled asthma, mental and behavioral problems, dental pain, persistent hunger, and the effects of lead exposure?

To assess the degree to which this was accomplished, investigators asked:

1. Does the state mandate comprehensive school health examinations?
  - If a school health examination is required, is it required at school entry only or more frequently?
  - If school health examination is required by legislation, is there a specific form that is required?
  - If a specific form is required, to what extent does the form include the 7 Health Barriers to Learning?
2. Does the state mandate schools to require students to be screened for vision, hearing and/or dental problems for school entry or more frequently? What is the required frequency?

### Comprehensive school health examinations

For information on requirements for comprehensive school health examinations, investigators reviewed state department of health and state department of education websites and also websites on state legislation for all 50 states and the District of Columbia (DC). For states where mandates for comprehensive school health examinations could not be found through any of these sources, attempts were made to contact both the departments of health and education via email and phone. In cases where none of these steps led to definitive identification of legislation or requirement for a form, investigators have noted that the information was not identified, versus a definitive conclusion that either or both do not exist.

### School health form content

For states that required a comprehensive school health examination and a specific school health form, investigators applied structured content analysis to the forms to assess inclusion of each HBL. Investigators identified key variables and assigned categorical values to cover the range of possible responses. For the purposes of this study, asthma was considered to be included on a form even if not specifying control status. Mental and behavioral problems were considered to be included if the form asked about general mental or behavioral health, specific mental health or behavioral conditions, or general well-being. Dental pain was included if the form required a dental exam or asked about dental problems. Persistent hunger was included if the form mentioned hunger or food insecurity.

Two investigators each coded all forms to determine inter-rater reliability. Any differing responses were discussed and resolved by consensus. Cohen’s Kappa yielded a score of 0.962 and there was 97.6% agreement. Responses were then analyzed using SPSS version 23 to determine descriptive frequencies. It should be noted that the high level of agreement most likely was influenced by the fact that both coders, though working independently, had worked with the research team to create the instrument, which increased familiarity. In addition, the coding was not subjective, it was a simple yes or no as to whether or not the information appeared on the form.

#### State-mandated screening requirements for vision, hearing and dental problems

Investigators conducted a preliminary review of the school requirements for screening of health conditions listed in the State School Health Policy Database compiled by the National Association of State Boards of Education. Among the Health Barriers to Learning, state mandates for screening vision, hearing and dental problems were the most common. Most states’ requirements for the screening or assessment of the other Health Barriers to Learning (uncontrolled asthma, effects of lead exposure, and mental health and behavioral problems and persistent hunger) were most often addressed through the comprehensive health examinations, if at all [64]. The research team focused on further investigating statewide screening requirements for vision, hearing, and dental problems through a thorough review of reports and websites compiling health-screening requirements by state [68–70].

The study was not submitted for review, as the Institutional Review Board at William Paterson University does not review studies that do not involve human subjects.

## Results

Information was obtained for 50 states and DC (see S1 Appendix for detailed state by state findings).

### Requirements for comprehensive health examinations

Only DC was found to require an annual comprehensive health examination for school. Other than DC, 24 states have legislation requiring comprehensive health examinations at school entry. Some were required more frequently, but not annually. For 26 states (51%), mandates for comprehensive school health examinations could not be identified (Table 1).

**Table 1:** Student health screening form requirements for states with and without required comprehensive school health examinations.

Form Requirement	Comprehensive Health Examination Required n = 25	Comprehensive Health Examination Not Required n = 26	Total N = 51
Required Form	13 (52%)	0 (0%)	13 (26%)
Form available but not required OR unclear if required	12 (48%)	7 (27%)	19 (37%)
No school health form	0 (0%)	19 (73%)	19 (37%)

### Requirements for school forms

For 12 states and DC, investigators identified language on the state website or in state legislation explicitly stating that a particular form or a form that is equivalent in content is required. For the remaining 12 states, the form is either optional or it is not clear whether the form or its contents are required or optional (Table 1).

### Required forms: Health Barriers to Learning

The 13 forms that were clearly required, 12 states (24% plus DC) were assessed for content. None included all 7 Health Barriers to Learning (Table 2). Twelve forms required documentation of vision screening, and 11 required documentation of hearing screening. Forms from 12 states required a dental screening (by a primary care or dental provider), and 9 asked about lead exposure. Ten forms asked if the student had diagnosed asthma but only four asked about general breathing issues, coughing, or wheezing. Nine of the forms asked about behavioral issues, and seven asked about general well-being, emotional problems, or general mental health. None of the forms inquired about or assessed risk for hunger.

**Table 2:** Inclusion of specific health areas on school forms from 12 states and D.C.

	Vision	Hearing	Dental	Asthma	Mental Health/ Behavior	Lead	Hunger
**California**	✓	✓	✓			✓	
**Connecticut**	✓	✓	✓	✓	✓	✓	
**D.C.**	✓	✓	✓	✓	✓	✓	
**Georgia**	✓	✓	✓				
**Hawaii**	✓	✓	✓	✓	✓		
**Illinois**	✓	✓	✓	✓	✓	✓	
**Kansas**	✓	✓	✓	✓	✓	✓	
**Kentucky**	✓	✓	✓		✓		
**Maryland**			✓	✓	✓	✓	
**Massachusetts**	✓	✓	✓	✓	✓	✓	
**North Carolina**	✓	✓	✓	✓	✓	✓	
**Pennsylvania**	✓	✓	✓	✓	✓		
**Rhode Island**	✓			✓	✓	✓	

#### State mandated school screening requirements for vision, hearing, and dental problems

Screening can be offered directly at the schools, or by requiring parents to obtain screenings from a child’s healthcare provider. Investigators identified 42 states (including DC, 82%) with vision screening requirements, and 38 states (including DC, 75%) with hearing screening requirements. Required frequency of screening varied by state, with only 4 states (including DC, 8%) identified with annual vision screening requirements and only DC with annual hearing screening. Dental screening requirements were less common, with requirements identified in only 12 states (including DC, 24%). See S1 Dataset for greater detail.

## Discussion

This study identified no state mandated assessment of all 7 Health Barriers to Learning for school records or participation. Annual comprehensive health screening requirements for school were only identified for DC. As presented in the results above, though most states (80%) do have student vision screening requirements and about 3/4 (75%) require hearing screening at least once, less than half (49%) require a comprehensive health examination.

Screening for HBLs is only one element of ensuring good health to optimize each child’s well being and experience in school. Ideally, all children should get their annual well child check as part of ongoing care from a medical home—a usual source of comprehensive, coordinated, high quality of care in their community. This should include developmental screening, sleep and trauma history, discussion of absences due to illness, assessment of school readiness, and identification and management of any disabilities or chronic illnesses that will require support in school. However, as mentioned in the introduction, 19% of children aged 6 to 11 did not receive their annual checkup in the past year, with higher rates for children in certain subpopulations, such as those who are uninsured (up to 44%) [60]. A school requirement for a health screening and physical examination form is one way to help ensure that families do go to community clinics to receive this care. The presence of specific elements on the form can guide clinicians to implement best-practice, age-appropriate screening protocols for each of the HBLs, ensure inclusion of elements most important to school performance in their examination, and facilitate communication of relevant findings to the school. Requirements for student health screening forms that include a comprehensive health examination have the added benefit of increasing the likelihood that children will receive other important health care, health education, and anticipatory guidance. Requirements for an ***annual*** review of age-appropriate screenings could help to ensure that health problems that develop and change over time are consistently identified and managed.

Access to community-based care remains challenging for many families for reasons of cost, transportation, inability to take off work, and competing priorities [71]. Partnerships with local health care providers and school-based screenings and services can help to ensure that children with these barriers are still appropriately screened and managed [72,73]. From a public health and cost standpoint, there are obviously limitations to what a school or school system can do in terms of direct screening and follow-up, but investment in this capacity may have potential for return on investment through reduced absenteeism, reduced grade-repetition, improved reading and math scores, and increased graduation rates. Legislation and appropriate funding will both be necessary for schools to be able to effectively offer such services for the students that need them. This will be especially important to schools and communities with large percentages of children living in poverty, as typically they will have the highest need, poorest healthcare access, and the least resources.

### Study limitations and need for further research

Information on requirements of each state was limited to what was publicly available and discoverable through the search methodology of the study. Additionally, this study focuses on state mandates and does not capture local-level requirements. Finally, it is important to note that existence of state mandates and specific forms does not equal compliance. Further research is recommended to explore compliance, and also to explore the extent to which school systems require and track resolution of health problems identified through screenings. Further cost-effectiveness, return on investment, and implementation strategies research would also facilitate evidence-based recommendations around screening, in particular for those offered directly in school. Further study of systems with successful implementation of annual student health screening requirements, like DC, could help to develop implementation and sustainability strategies.

## Conclusions

The lack of state requirements for annual health screening of students in elementary schools represents a missed opportunity to identify children who are burdened with HBLs. Without state mandates and accompanying comprehensive forms and protocols, children continue to be at risk of untreated health conditions that undermine their success in school.

## Supporting information

S1 Appendix State-by-state requirements for school screenings in grades Pre-K to 6. (DOCX)

S1 Dataset Data for state- level school health screening requirements grades Pre-K to 6. (XLSX)

## References

[1] Case A, Fertig A, Paxson C. The lasting impact of childhood health and circumstance. J Health Econ. 2005;24: 365–389. doi: 10.1016/j.jhealeco.2004.09.008 15721050 10.1016/j.jhealeco.2004.09.008

[2] McLoyd VC. Socioeconomic disadvantage and child development. Am Psychol. 1998;53: 185–204. Available: https://www.ncbi.nlm.nih.gov/pubmed/9491747 9491747 10.1037//0003-066x.53.2.185

[3] Basch CE. Healthier students are better learners: a missing link in school reforms to close the achievement gap. J Sch Health. 2011;81: 593–598. doi: 10.1111/j.1746-1561.2011.00632.x 21923870 10.1111/j.1746-1561.2011.00632.x

[4] Ou S-R, Reynolds AJ. Predictors of educational attainment in the Chicago Longitudinal Study. Sch Psychol Q. Educational Publishing Foundation; 2008;23: 199 Available: http://psycnet.apa.org/journals/spq/23/2/199/

[5] Basch CE. Healthier students are better learners: a missing link in school reforms to close the achievement gap. J Sch Health. 2011;81: 593–598. doi: 10.1111/j.1746-1561.2011.00632.x 21923870 10.1111/j.1746-1561.2011.00632.x

[6] Michael SL, Merlo CL, Basch CE, Wentzel KR, Wechsler H. Critical connections: health and academics. J Sch Health. 2015;85: 740–758. doi: 10.1111/josh.12309 26440816 10.1111/josh.12309 PMC4606776

[7] Gracy D, Fabian A, Roncaglione V, Savage K, Redlener I. Health Barriers to Learning: The Prevalence and Educational Consequences in Disadvantaged Children [Internet]. Children’s Health Fund; 2017 1 Available: https://www.childrenshealthfund.org/hbl-literature-review/

[8] Bodack MI, Chung I, Krumholtz I. An analysis of vision screening data from New York City public schools. Optometry. 2010;81: 476–484. doi: 10.1016/j.optm.2010.05.006 20619746 10.1016/j.optm.2010.05.006

[9] Krumholtz I. Results from a pediatric vision screening and its ability to predict academic performance. Optometry. 2000;71: 426–430. Available: https://www.ncbi.nlm.nih.gov/pubmed/15326895 15326895

[10] Choi TB, Lee DA, Oelrich FO, Amponash D, Bateman JB, Christensen RE. A retrospective study of eye disease among first grade children in Los Angeles. J Am Optom Assoc. 1995;66: 484–488. Available: https://www.ncbi.nlm.nih.gov/pubmed/7494083 7494083

[11] Pizzarello L, Tilp M, Tiezzi L, Vaughn R, McCarthy J. A new school-based program to provide eyeglasses: childsight. J AAPOS. 1998;2: 372–374. Available: https://www.ncbi.nlm.nih.gov/pubmed/10532728 10532728 10.1016/s1091-8531(98)90038-6

[12] Basch CE. Vision and the achievement gap among urban minority youth. J Sch Health. 2011;81: 599–605. doi: 10.1111/j.1746-1561.2011.00633.x 21923871 10.1111/j.1746-1561.2011.00633.x

[13] VIP-HIP Study Group, Kulp MT, Ciner E, Maguire M, Moore B, Pentimonti J, et al Uncorrected Hyperopia and Preschool Early Literacy: Results of the Vision in Preschoolers-Hyperopia in Preschoolers (VIP-HIP) Study. Ophthalmology. 2016;123: 681–689. doi: 10.1016/j.ophtha.2015.11.023 26826748 10.1016/j.ophtha.2015.11.023 PMC4808323

[14] Barnhardt C, Borsting E, Deland P, Pham N, Vu T. Relationship between visual-motor integration and spatial organization of written language and math. Optom Vis Sci. 2005;82: 138–143. Available: https://www.ncbi.nlm.nih.gov/pubmed/15711461 15711461 10.1097/01.opx.0000153266.50875.53

[15] Bright K, Eichwald J, Hall J, Hanks WD, Ingrao B, Mauceri P, et al American Academy of Audiology Childhood Hearing Screening Guidelines [Internet]. American Academy of Audiology; 2011 9 Available: https://www.cdc.gov/ncbddd/hearingloss/documents/aaa_childhood-hearing-guidelines_2011.pdf

[16] Sekhar DL, Zalewski TR, Beiler JS, Czarnecki B, Barr AL, King TS, et al The Sensitivity of Adolescent Hearing Screens Significantly Improves by Adding High Frequencies. J Adolesc Health. 2016;59: 362–364. doi: 10.1016/j.jadohealth.2016.02.002 27021402 10.1016/j.jadohealth.2016.02.002

[17] Bess FH, Dodd-Murphy J, Parker RA. Children with minimal sensorineural hearing loss: prevalence, educational performance, and functional status. Ear Hear. 1998;19: 339–354. Available: https://www.ncbi.nlm.nih.gov/pubmed/9796643 9796643 10.1097/00003446-199810000-00001

[18] Lieu JEC. Speech-language and educational consequences of unilateral hearing loss in children. Arch Otolaryngol Head Neck Surg. 2004;130: 524–530. doi: 10.1001/archotol.130.5.524 15148171 10.1001/archotol.130.5.524

[19] Lieu JEC, Tye-Murray N, Karzon RK, Piccirillo JF. Unilateral hearing loss is associated with worse speech-language scores in children. Pediatrics. 2010;125: e1348–55. doi: 10.1542/peds.2009-2448 20457680 10.1542/peds.2009-2448 PMC3469199

[20] National Center for Environmental Health. Current Asthma Prevalence Percents by Age, United States: National Health Interview Survey, 2015 [Internet]. Centers for Disease Control; 2017 Available: https://www.cdc.gov/asthma/nhis/2015/table4-1.htm

[21] Halterman JS, Montes G, Aligne CA, Kaczorowski JM, Hightower AD, Szilagyi PG. School readiness among urban children with asthma. Ambul Pediatr. 2001;1: 201–205. Available: https://www.ncbi.nlm.nih.gov/pubmed/11888401 11888401 10.1367/1539-4409(2001)001<0201:sraucw>2.0.co;2

[22] Taras H, Potts-Datema W. Childhood asthma and student performance at school. J Sch Health. 2005;75: 296–312. doi: 10.1111/j.1746-1561.2005.00041.x 16179080 10.1111/j.1746-1561.2005.00041.x

[23] Diette GB, Markson L, Skinner EA, Nguyen TT, Algatt-Bergstrom P, Wu AW. Nocturnal asthma in children affects school attendance, school performance, and parents’ work attendance. Arch Pediatr Adolesc Med. 2000;154: 923–928. Available: https://www.ncbi.nlm.nih.gov/pubmed/10980797 10980797 10.1001/archpedi.154.9.923

[24] Szefler SJ, Zeiger RS, Haselkorn T, Mink DR, Kamath TV, Fish JE, et al Economic burden of impairment in children with severe or difficult-to-treat asthma. Ann Allergy Asthma Immunol. 2011;107: 110–119.e1. doi: 10.1016/j.anai.2011.04.008 21802018 10.1016/j.anai.2011.04.008

[25] National Center for Environmental Health. Number and Percent of Asthma-related Missed School Days among Children aged 5–17 Years: United States, 2003, 2008, 2013 [Internet]. Centers for Disease Control; 2015 Available: https://www.cdc.gov/asthma/asthma_stats/missing_days.htm

[26] Henning-Smith C, Alang S. Access to care for children with emotional/behavioral difficulties. J Child Health Care. 2016;20: 185–194. doi: 10.1177/1367493514563855 25583944 10.1177/1367493514563855 PMC4499501

[27] Simon AE, Pastor PN, Reuben CA, Huang LN, Goldstrom ID. Use of Mental Health Services by Children Ages Six to 11 With Emotional or Behavioral Difficulties. Psychiatr Serv. 2015;66: 930–937. doi: 10.1176/appi.ps.201400342 25975889 10.1176/appi.ps.201400342 PMC4654459

[28] Esch P, Bocquet V, Pull C, Couffignal S, Lehnert T, Graas M, et al The downward spiral of mental disorders and educational attainment: a systematic review on early school leaving. BMC Psychiatry. 2014;14: 237 doi: 10.1186/s12888-014-0237-4 25159271 10.1186/s12888-014-0237-4 PMC4244046

[29] Breslau J, Lane M, Sampson N, Kessler RC. Mental disorders and subsequent educational attainment in a US national sample. J Psychiatr Res. 2008;42: 708–716. doi: 10.1016/j.jpsychires.2008.01.016 18331741 10.1016/j.jpsychires.2008.01.016 PMC2748981

[30] Subcommittee on Attention-Deficit/Hyperactivity Disorder, Steering Committee on Quality Improvement and Management, Wolraich M, Brown L, Brown RT, DuPaul G, et al ADHD: clinical practice guideline for the diagnosis, evaluation, and treatment of attention-deficit/hyperactivity disorder in children and adolescents. Pediatrics. 2011;128: 1007–1022. doi: 10.1542/peds.2011-2654 22003063 10.1542/peds.2011-2654 PMC4500647

[31] Centers for Disease Control. National Health and Nutrition Examination Survey (NHANES): Children with dental caries experience in their primary or permanent teeth (percent, 6–9 years) [Internet] U.S. Department of Health and Human Services; 2015 Available: https://www.healthypeople.gov/2020/data/disparities/summary/Chart/4993/6.1

[32] Jackson SL, Vann WF Jr, Kotch JB, Pahel BT, Lee JY. Impact of poor oral health on children’s school attendance and performance. Am J Public Health. 2011;101: 1900–1906. doi: 10.2105/AJPH.2010.200915 21330579 10.2105/AJPH.2010.200915 PMC3222359

[33] More vigilance needed in children’s oral health [Internet]. [cited 19 Sep 2017]. Available: http://www.ur.umich.edu/0506/Feb06_06/20.shtml

[34] Seirawan H, Faust S, Mulligan R. The impact of oral health on the academic performance of disadvantaged children. Am J Public Health. 2012;102: 1729–1734. doi: 10.2105/AJPH.2011.300478 22813093 10.2105/AJPH.2011.300478 PMC3482021

[35] Pourat N, Nicholson G. Unaffordable Dental Care Is Linked to Frequent School Absences (Los Angeles, CA: UCLA Center for Health Policy Research, 2009), 1–6. Expanding California’s Dental Team to Care for Underserved Children: New Times, New Solutions Expanding California’s Dental Team to Care for Underserved Children: New Times, New Solutions. 19960616

[36] Coleman-Jensen A, Rabbitt M, Gregory C, Singh A. Household Food Security in the United States in 2015, ERR-215 (Washington, DC: US Department of Agriculture, Economic Research Service, 9 2016). https://www.ers.usda.gov/webdocs/publications/79761/err-215.pdf

[37] Alaimo K, Olson CM, Frongillo EA Jr. Food insufficiency and American school-aged children’s cognitive, academic, and psychosocial development. Pediatrics. 2001;108: 44–53. Available: https://www.ncbi.nlm.nih.gov/pubmed/11433053 11433053

[38] Jyoti DF, Frongillo EA, Jones SJ. Food insecurity affects school children’s academic performance, weight gain, and social skills. J Nutr. 2005;135: 2831–2839. Available: https://www.ncbi.nlm.nih.gov/pubmed/16317128 16317128 10.1093/jn/135.12.2831

[39] Ashiabi G. Household food insecurity and children’s school engagement. Journal of Children and Poverty. Routledge; 2005;11: 3–17. doi: 10.1080/1079612042000333027

[40] Winicki J, Jemison K. Food Insecurity and Hunger in the Kindergarten Classroom: Its Effect on Learning and Growth. Contemp Econ Policy. Blackwell Publishing Ltd; 2003;21: 145–157. doi: 10.1093/cep/byg001

[41] Kleinman RE, Murphy JM, Little M, Pagano M, Wehler CA, Regal K, et al Hunger in children in the United States: potential behavioral and emotional correlates. Pediatrics. 1998;101: E3 Available: https://www.ncbi.nlm.nih.gov/pubmed/9417167 10.1542/peds.101.1.e3 9417167

[42] Centers for Disease Control and Prevention (CDC). Blood lead levels in children aged 1–5 years—United States, 1999–2010. MMWR Morb Mortal Wkly Rep. 2013;62: 245–248. Available: https://www.ncbi.nlm.nih.gov/pubmed/23552225 23552225 PMC4605011

[43] McLaine P, Navas-Acien A, Lee R, Simon P, Diener-West M, Agnew J. Elevated blood lead levels and reading readiness at the start of kindergarten. Pediatrics. 2013;131: 1081–1089. doi: 10.1542/peds.2012-2277 23669514 10.1542/peds.2012-2277

[44] Advisory Committee on Childhood Lead Poisoning Prevention. Low Level Lead Exposure Harms Children: A Renewed Call for Primary Prevention [Internet]. Centers for Disease Control and Prevention; 2012 1 Available: https://www.cdc.gov/nceh/lead/acclpp/final_document_030712.pdf

[45] Lanphear BP, Dietrich K, Auinger P, Cox C. Cognitive deficits associated with blood lead concentrations <10 microg/dL in US children and adolescents. Public Health Rep. 2000;115: 521–529. Available: https://www.ncbi.nlm.nih.gov/pubmed/11354334 11354334 10.1093/phr/115.6.521 PMC1308622

[46] Miranda ML, Kim D, Reiter J, Overstreet Galeano MA, Maxson P. Environmental contributors to the achievement gap. Neurotoxicology. 2009;30: 1019–1024. doi: 10.1016/j.neuro.2009.07.012 19643133 10.1016/j.neuro.2009.07.012 PMC2789840

[47] Lanphear BP, Hornung R, Khoury J, Yolton K, Baghurst P, Bellinger DC, et al Low-level environmental lead exposure and children’s intellectual function: an international pooled analysis. Environ Health Perspect. 2005;113: 894–899. Available: https://www.ncbi.nlm.nih.gov/pubmed/16002379 doi: 10.1289/ehp.7688 16002379 10.1289/ehp.7688 PMC1257652

[48] Evens A, Hryhorczuk D, Lanphear BP, Rankin KM, Lewis DA, Forst L, et al The impact of low-level lead toxicity on school performance among children in the Chicago Public Schools: a population-based retrospective cohort study. Environ Health. 2015;14: 21 doi: 10.1186/s12940-015-0008-9 25889033 10.1186/s12940-015-0008-9 PMC4387706

[49] Kerker BD, Zhang J, Nadeem E, Stein REK, Hurlburt MS, Heneghan A, et al Adverse Childhood Experiences and Mental Health, Chronic Medical Conditions, and Development in Young Children. Acad Pediatr. 2015;15: 510–517. doi: 10.1016/j.acap.2015.05.005 26183001 10.1016/j.acap.2015.05.005 PMC4562867

[50] Bethell CD, Newacheck P, Hawes E, Halfon N. Adverse childhood experiences: assessing the impact on health and school engagement and the mitigating role of resilience. Health Aff. 2014;33: 2106–2115. doi: 10.1377/hlthaff.2014.0914 25489028 10.1377/hlthaff.2014.0914

[51] Blodgett C, Lanigan J, Harrington R, Lohan J, Short R, Turner N, et al Adverse childhood experience and developmental risk in elementary schoolchildren. Manuscript in preparation. 2014;

[52] Dewald JF, Meijer AM, Oort FJ, Kerkhof GA, Bögels SM. The influence of sleep quality, sleep duration and sleepiness on school performance in children and adolescents: A meta-analytic review. Sleep Med Rev. 2010;14: 179–189. doi: 10.1016/j.smrv.2009.10.004 20093054 10.1016/j.smrv.2009.10.004

[53] Meijer AM, van den Wittenboer GLH. The joint contribution of sleep, intelligence and motivation to school performance. Pers Individ Dif. 2004;37: 95–106. Available: http://www.sciencedirect.com/science/article/pii/S0191886903003325

[54] Bright Futures/American Academy of Pediatrics. In: Recommendations for Preventive Pediatric Health Care [Internet]. 2017. Available: https://www.aap.org/en-us/Documents/periodicity_schedule.pdf

[55] Council on Community Pediatrics, Committee on Nutrition. Promoting Food Security for All Children. Pediatrics. 2015;136: e1431–8. doi: 10.1542/peds.2015-3301 26498462 10.1542/peds.2015-3301

[56] Bright Futures, American Academy of Pediatrics. Recommendations for Preventive Pediatric Health Care [Internet]. American Academy of Pediatrics; 2017 4 Available: https://www.aap.org/en-us/Documents/periodicity_schedule.pdf

[57] Third Expert Panel on the Management of Asthma, National Asthma Education and Prevention Program Coordinating Committee. National Asthma Education and Prevention Program Expert Panel Report 3: Guidelines for the Diagnosis and Management of Asthma Section3, The Four Components of Asthma Management [Internet]. U.S. Department of Health and Human Services National Institutes of Health; 2007 8 Report No.: 3. Available: https://www.nhlbi.nih.gov/files/docs/guidelines/04_sec3_comp.pdf

[58] Council on Community Pediatrics. Poverty and Child Health in the United States. Pediatrics. 2016;137 doi: 10.1542/peds.2016-0339 26962238 10.1542/peds.2016-0339

[59] Hagan JF, Shaw JS, Duncan PM. Bright Futures, 3rd Edition [Internet]. American Academy of Pediatrics; 2007 Available: http://ebooks.aappublications.org/content/bright-futures-3rd-edition

[60] National Health Interview Survey-Child and Family Core, NHIS-Child. Data query from the Child and Adolescent Health Measurement Initiative, Data Resource Center for Child and Adolescent Health website. In: Data Resource Center for Child and Adolescent Health website. [Internet]. 2014 [cited 15 Mar 2017]. Available: www.childhealthdata.org

[61] Basch CE, Gracy D, Johnson D, Fabian A. Health Barriers to Learning and the Education Opportunity Gap Progress of Education Reform [Internet]. Education Commission of the States; 2015 Available: http://www.ecs.org/clearinghouse/01/20/69/12069.pdf

[62] Brandon RN, Stahr-Breunig G. Healthy to Learn: State Requirements for Child Health Examinations Human Services Policy Center, Evans School of Public Affairs, University of Washington; 2001.

[63] Sekhar DL, Zalewski TR, Paul IM. Variability of state school-based hearing screening protocols in the United States. J Community Health. 2013;38: 569–574. doi: 10.1007/s10900-013-9652-6 23355103 10.1007/s10900-013-9652-6

[64] NASBE State School Health Policy Database. In: NASBE State School Health Policy Database [Internet] National Association of State Boards of Education; Available:

[65] Mensah GA, Goodman RA, Zaza S, Moulton AD, Kocher PL, Dietz WH, et al Law as a tool for preventing chronic diseases: expanding the range of effective public health strategies. Prev Chronic Dis. 2004;1: A13 Available: https://www.ncbi.nlm.nih.gov/pubmed/15634375 PMC544536 15634375

[66] Green DR, Gaffney M, Devine O, Grosse SD. Determining the effect of newborn hearing screening legislation: an analysis of state hearing screening rates. Public Health Rep. 2007;122: 198–205. doi: 10.1177/003335490712200209 17357362 10.1177/003335490712200209 PMC1820423

[67] Asthma and Allergy Foundation of America. 2016 State Honor Roll Report: Asthma Allergy Policies in Schools [Internet] Landover, MD: Asthma and Allergy Foundation of America; Available: http://www.aafa.org/media/2016-State-Honor-Roll-Report-Asthma-Allergy-Policies-in-Schools.pdf

[68] Ruderman M. Children’s Vision and Eye Health: A Snapshot of Current National Issues (1st ed) [Internet]. National Center for Children’s Vision and Eye Health at Prevent Blindness; 2016 Available: http://nationalcenter.preventblindness.org/sites/default/files/national/documents/Childrens_Vision_Chartbook_F.pdf

[69] American Speech-Language-Hearing Association (ASHA). Hearing screening requirements by state compiled by American Speech-Language-Hearing Association (ASHA). In: Hearing screening requirements by state compiled by American Speech-Language-Hearing Association (ASHA) [Internet]. Available: http://www.asha.org/advocacy/state/

[70] Booth M, Frosh M. State Laws on Dental “Screening” for School-Aged Children [Internet]. Children’s Dental Health Project; Association of State and Territorial Dental Directors; 2008 10 Available: http://www.astdd.org/docs/final-school-screening-paper-10-14-08-9-21-2015-edits.pdf

[71] Redlener I, Gracy D, Walto D, Sobel C, Fabian A, Roncaglione V. Unfinished Business: More than 20 Million Children in U.S. Still Lack Sufficient Access to Essential Health Care [Internet]. Children’s Health Fund; 2016 11 Available: https://www.childrenshealthfund.org/wp-content/uploads/2016/11/Unfinished-Business-Final_.pdf

[72] Preslan MW, Novak A. Baltimore Vision Screening Project. Ophthalmology. 1996;103: 105–109. doi: 10.1016/s0161-6420(96)30753-7 8628540 10.1016/s0161-6420(96)30753-7

[73] Locker D, Frosina C, Murray H, Wiebe D, Wiebe P. Identifying children with dental care needs: evaluation of a targeted school-based dental screening program. J Public Health Dent. 2004;64: 63–70. Available: https://www.ncbi.nlm.nih.gov/pubmed/15180073 15180073 10.1111/j.1752-7325.2004.tb02729.x

