# Supplementary material for: Missed opportunities: Do states require screening of children for health conditions that interfere with learning?
Source: PLoS One. 2018 Jan 17;13(1):e0190254. doi: 10.1371/journal.pone.0190254 (PMC5771574; doi:10.1371/journal.pone.0190254)
Supplement: S1 Appendix — (DOCX) [file pone.0190254.s001.docx]

**S1. Appendix: Table showing state-by-state requirements for school screenings in grades Pre-K to 6**

| **State** | **Legislation Exists Requiring Comprehensive Health Exam for School Entry** | **Frequency of Required School Health Exam** | **State Form**  *Recommended **Required | **Vision Screening Required** | **Vision Screening Frequency** | **Hearing Screening Required** | **Hearing Screening Frequency** | **Dental Screening Required** |
| --- | --- | --- | --- | --- | --- | --- | --- | --- |
| Alabama | - | N/A | * | - | N/A | - | N/A | - |
| Alaska | ✓ | 1 | * | ✓ | 1 | ✓ | 1 | - |
| Arizona | - | N/A | - | - | N/A | ✓ | 3 | - |
| Arkansas | ✓ | 1 | * | ✓ | 6 | ✓ | 6 | - |
| California | ✓ | 1 | ** | ✓ | 3 | ✓ | 4 | ✓ |
| Colorado | - | N/A | - | ✓ | 5 | ✓ | 6 | - |
| Connecticut | ✓ | 1 | ** | ✓ | 7 | ✓ | 5 | - |
| D.C. | ✓ | 8 | ** | ✓ | 8 | ✓ | 8 | ✓ |
| Delaware | ✓ | 1 | * | ✓ | 3 | ✓ | 3 | - |
| Florida | ✓ | 1 | * | ✓ | 4 | ✓ | 3 | - |
| Georgia | ✓ | 1 | ** | ✓ | 1 | ✓ | 2 | ✓ |
| Hawaii | ✓ | 1 | ** | ✓ | 1 | ✓ | 1 | - |
| Idaho | - | N/A | - | - | N/A | - | 1 | - |
| Illinois | ✓ | 3 | ** | ✓ | 4 | ✓ | 5 | ✓ |
| Indiana | - | N/A | - | ✓ | 3 | ✓ | 2 | - |
| Iowa | - | N/A | - | ✓ | 2 | - | N/A | ✓ |
| Kansas | ✓ | 1 | ** | ✓ | 3 | ✓ | 3 | ✓ |
| Kentucky | ✓ | 2 | ** | ✓ | 2 | ✓ | 2 | ✓ |
| Louisiana | - | N/A | * | ✓ | 4 | ✓ | 1 | - |
| Maine | - | N/A | * | ✓ | 4 | ✓ | 4 | - |
| Maryland | ✓ | 1 | ** | ✓ | 2 | ✓ | 2 | - |
| Massachusetts | ✓ | 2 | ** | ✓ | 7 | ✓ | 5 | - |
| Michigan | - | N/A | * | ✓ | 4 | ✓ | 3 | - |
| Minnesota | ✓ | 1 | * | ✓ | 4 | ✓ | 1 | - |
| Mississippi | - | N/A | - | ✓ | Unclear | ✓ | Unclear | - |
| Missouri | - | N/A | - | ✓ | 2 | - | N/A | - |
| Montana | - | N/A | - | - | N/A | ✓ | 2 | - |
| Nebraska | ✓ | 1 | * | ✓ | 6 | ✓ | 6 | ✓ |
| Nevada | - | N/A | - | ✓ | 2 | ✓ | 2 | - |
| New Hampshire | ✓ | 1 | * | - | N/A | - | N/A | - |
| New Jersey | ✓ | 1 | * | ✓ | 3 | ✓ | 4 | - |
| New Mexico | - | N/A | * | ✓ | 4 | - | N/A | - |
| New York | ✓ | 4 | * | ✓ | 6 | ✓ | 5 | ✓ |
| North Carolina | ✓ | 1 | ** | ✓ | 1 | ✓ | 1 | - |
| North Dakota | - | N/A | - | - | N/A | - | N/A | - |
| Ohio | ✓ | 1 | * | ✓ | 4 | ✓ | 5 | - |
| Oklahoma | - | N/A | - | ✓ | 3 | - | N/A | - |
| Oregon | - | N/A | * | ✓ | 4 | - | N/A | ✓ |
| Pennsylvania | ✓ | 2 | ** | ✓ | 8 | ✓ | 4 | ✓ |
| Rhode Island | ✓ | 1 | ** | ✓ | 6 | ✓ | 5 | ✓ |
| South Carolina | - | N/A | - | - | N/A | - | N/A | - |
| South Dakota | - | N/A | - | - | N/A | - | N/A | - |
| Tennessee | - | N/A | - | ✓ | 5 | Unclear | N/A | - |
| Texas | - | N/A | - | ✓ | 5 | ✓ | 5 | - |
| Utah | - | N/A | * | ✓ | 1 | - | N/A | - |
| Vermont | - | N/A | - | ✓ | 4 | ✓ | 4 | - |
| Virginia | ✓ | 1 | * | ✓ | 3 | ✓ | 2 | - |
| Washington | - | N/A | - | ✓ | 4 | ✓ | 5 | - |
| West Virginia | ✓ | 1 | * | ✓ | 1 | ✓ | 1 | - |
| Wisconsin | - | N/A | - | - | N/A | - | N/A | - |
| Wyoming | - | N/A | - | - | N/A | - | N/A | - |

✓, Yes; -, Legislation could not be found; N/A, Not Applicable;
